# Successful outcome after Pancreaticodoudenectomy in an elderly cirrhotic patient: A case report

**DOI:** 10.1016/j.ijscr.2019.03.034

**Published:** 2019-04-10

**Authors:** Eraj Sahaab, Nadeem Iqbal, Abu Bakar Hafeez Bhatti

**Affiliations:** a Department of HPB Surgery and Liver Transplantation, Shifa International Hospital, Islamabad, Pakistan; b Department of Gastroenterlogy and Hepatology, Shifa International Hospital, Islamabad, Pakistan; c Department of Surgery, Shifa Tameer-e-Millat University, Islamabad, Pakistan

**Keywords:** Liver cirrhosis, Whipple's procedure, Child Pugh Score, Morbidity

## Abstract

**Introduction:**

Cirrhosis is a risk factor for poor outcomes in non-hepatic oncological resections. There are only a handful of cases demonstrating outcomes of pancreaticoduodenectomy (PD) in patients with pancreatic cancer and underlying cirrhosis. Moreover, pushing the limits of PD to elderly cirrhotic patients and demonstrating its safety remains under reported.

**Case presentation:**

Here, we report successful management of a 71 year-old-lady with HCV related liver cirrhosis, obstructive jaundice and poor performance status, who was medically optimized and then underwent PD for adenocarcinoma of the head of pancreas. There was no major post-operative morbidity except minor chyle leak which resolved spontaneously. On her last follow up, she was doing well and undergoing adjuvant chemotherapy.

**Discussion:**

Preoperative medical optimization and careful patient selection can yield successful outcomes even after a major abdominal surgery like PD in elderly patients with underlying liver cirrhosis.

**Conclusion:**

Liver cirrhosis is not a contra indication to PD in elderly patients with good performance status.

## Highlights

• Pancreaticodoudenectomy in cirrhotic patients is seldom attempted.
• It is associated with high morbidity and mortality rates.
• Careful patient selection allows its safe performance in cirrhotic patients.
• Preoperative biliary drainages should always be considered.
• Liver cirrhosis is not an absolute contraindication to PD.

## 1 Introduction

Surgical resection remains the only curative option for patients with resectable pancreatic adenocarcinoma. Presence of liver cirrhosis increases the risk of adverse post operative outcomes [1,2]. Old age and MELD score is an important risk factor for post-operative morbidity and mortality in these patients [3]. There is lack of data on the safety of PD in elderly cirrhotic patients. The model for end stage liver disease (MELD) has not been incorporated in majority of available literature on PD in cirrhotics. Moreover, assessment of liver failure in these patients who are otherwise at risk of hyperbilirubinemia and deranged coagulation profile due to obstructive jaundice remains unclear. Here, the authors have reported successful outcome after PD in a 71-year-old cirrhotic patient with diagnosis of adenocarcinoma of the head of the pancreas.

The current work has been reported in line with the SCARE criteria [4].

## 2 Case Study

A 71 year-old-lady with past history of hepatitis C virus (HCV) related cirrhosis was referred to the surgical clinic for obstructive jaundice. Her associated complaints included abdominal pain along with intermittent high grade fever. There were no co-morbids and no past surgical history. At the time of her presentation, her BMI was 21 kg/m^2^ and performance status (Eastern co operative oncology group (ECOG score) was 3). A CT scan with pancreatic protocol confirmed the presence of resectable pancreatic tumor. She was discussed in the multi disciplinary team meeting and was referred for endoscopic retrograde cholangiopancreatography (ERCP), endoscopic ultrasound(EUS) and HCV treatment followed by surgical resection if feasible. She underwent ERCP and stenting twice. Endoscopic ultrasound guided biopsy of pancreatic lesion was performed which showed atypical glandular proliferation suspicious of adenocarcinoma. Since a period of 4–6 weeks is recommended between ERCP and surgery, she was also started on direct acting anti virals due to high viral load. Her labs before and after biliary drainage and HCV treatment are shown in Table 1.

**Table 1:** Biochemical profile and functional status before and after biliary drainage and HCV treatment.

	Before Biliary drainage	4 weeks Post Biliary drainage
Total Bilirubin	13.8 mg/dl	1.2 mg/dl
Direct Bilirubin	9.3 mg/dl	0.8 mg/dl
AST	91 U/L	45 U/L
ALT	57 U/L	30 U/L
Alkaline Phosphatase	157 U/L	103 U/L
GGT	140 U/L	161 U/L
Serum Albumin	2.9	3.1
INR	1.2	1.2
MELD NA	23	9
CTP score	B (8)	A (6)
ECOG status	3	1

**Fig. 1:**
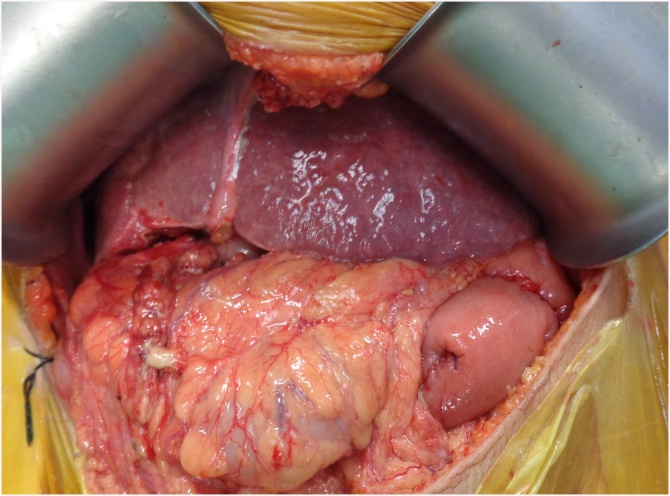
Macro nodular cirrhosis in HCV +ve patient undergoing pancreaticodoudenectomy.

**Fig. 2:**
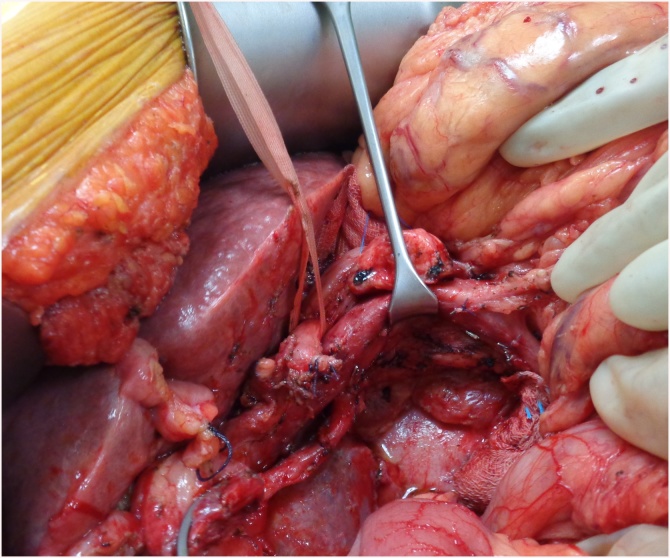
After specimen retrieval and portal lymphadenectomy. Note a replaced right hepatic artery from superior mesenteric artery.

Due to marked improvement in health, she was offered PD. Intra operatively, she had macro nodular liver cirrhosis (Fig. 1). She underwent PD with peri portal lymphadenectomy as shown in Fig. 2. A Roux loop was fashioned and end to side duct to mucosa pancreaticojejunostomy was performed with PDS 6/0. Intra operative blood loss was 200 ml. Operative time was 6 h. Post operative ICU stay was 4 days. She received Imipenem and Vancomycin for 5 days in the post operative period. Nasojejunal feeding was started on post operative day 2 while oral feed was resumed on day 3. Day 4 drain amylase was 9 U/L and 12 U/L. Her liver function tests and coagulation profile was performed daily for 3 days and was within normal range. The patient had a minor chyle leak which settled on its own with fat free diet. Due to underlying cirrhosis, the patient was kept on low salt diet and fluid restriction. The patient was discharged on 6th post operative day. The histopathology of resected specimen showed moderately differentiated pancreatic ductal adenocarcinoma (pT3N1) with peri neural and lymphovascular invasion. All margins were negative and one lymph node was positive for metastasis. The patient remains in good health three months after surgery and is receiving adjuvant chemotherapy at this time.

## 3 Discussion

Liver cirrhosis significantly increases the peri-operative morbidity and mortality following major surgeries [[1], [2], [3]]. In particular, safety of PD in cirrhotic patients has not been fully demonstrated. Moreover, the impact of old age in such patients has not been exclusively studied and reported.

There is limited literature on outcomes of PD in cirrhotic patients. These studies have used Child Turcot Pugh (CTP) score for assessment of liver failure and MELD score has not been utilized. Busquets and colleagues compared 15 patients with CTP A cirrhosis with a control group of 30 non-cirrhotic patients undergoing PD. Morbidity rate in cirrhotics was high (73%) [5].

It was not possible to determine if patients aged >70 years underwent PD in the cirrhotic group. It was also not shown whether degree of liver failure was assessed before or after biliary drainage. In another comparative study, El Nakeeb and colleagues showed increased operative time, blood loss, morbidity and mortality in cirrhotic patients. The study showed increased risk of bleeding in patients with liver cirrhosis (53.7% cirrhotics had blood loss >500 ml) [6]. They used a cut off of 10 mg/dl for biliary drainage. Around 37.3% cirrhotic patients did not have pre operative ERCP. This could have resulted in erroneous elevation of CTP scores. In our case, patient's MELD score dropped from 23 to 9 and CTP score from B(8) to A(6) after biliary drainage. This was associated with concomitant improvement in ECOG score. The patient had been denied surgical intervention elsewhere due to high MELD and CTP score and poor ECOG. This was however, falsely elevated due to obstructive jaundice. It is important that in these patients a low threshold for ERCP is kept since biliary drainage improves CTP and MELD score and overall health of the patient.

It has been shown that in cirrhotic patients, a single point increase in MELD score above 8 increases post operative mortality by 15% [3]. It has also been shown that age >70 years adds 3 points to the MELD score. Based on this assumption, our patient had a 60% mortality risk in the post operative period. The successful outcome in the current case was possible owing to careful pre-operative planning. It can be argued that in the presence of poor prognostic factors like cirrhosis, age >70 and MELD >8 and ECOG of 3; a non surgical palliative approach should have been considered in this patient. However excellent response to preoperative optimization with normalization of LFTs and improvement in ECOG after biliary drainage and HCV treatment signified possibility of a favorable outcome in this patient. It has been shown that high volume centers can have acceptable outcomes after PD even in challenging scenarios [7,8]. The authors believe that a high volume center, meticulous attention to surgical detail with minimal intra-operative blood loss, careful patient selection, use of broad spectrum antibiotics, salt and fluid restriction to prevent exacerbation of liver failure and simultaneous existence of an experienced liver transplant program [8] are some of the factors that had a positive impact on post-operative outcomes. We believe that routine total bilirubin cut off of 10 mg/dl should not be used in cirrhotic patients for biliary drainage since high bilirubin many a times is not because of liver failure but biliary obstruction. Attention to surgical detail, tailored post- operative care with focus on infection control, preemptive management of liver failure and early mobilization and initiation of enteral feeding might produce outcomes comparable to non-cirrhotic patients undergoing PD.

## 4 Conclusion

Liver cirrhosis should not be considered an absolute contraindication to PD. Pre-operative optimization in patients with low grade liver failure yielding good performance status produces acceptable outcomes even in elderly patients. Aggressive preoperative biliary drainage in the preoperative period and pre emptive management of liver failure post operatively improves outcomes after PD in cirrhotics.

## Conflict of interest

None of the authors have any conflicts of interests.

## Funding

No funding source.

## Ethical approval

This is a single case report and did not need ethical approval from hospital ethics committee.

## Consent

The patient gave informed consent fof the details of this case to be shared for academic purpose.

## Author contribution

Eraj Sahaab: concept, writing, critical review.

Nadeem Iqbal: Writing, critical review.

Abu Bakar Hafeez Bhatti: concept, writing, review.

## Registration of research studies

Not a first-in-man. Single case report. Registration not required.

## Guarantor

Abu bakar Hafeez Bhatti.

## Provenance and peer review

Not commissioned, externally peer-reviewed.
